# The Response of *Volvariella volvacea* to Low-Temperature Stress Based on Metabonomics

**DOI:** 10.3389/fmicb.2020.01787

**Published:** 2020-07-29

**Authors:** Xu Zhao, Mingjie Chen, Zhiping Li, Yan Zhao, Huanling Yang, Lei Zha, Changxia Yu, Yuejin Wu, Xiaoxia Song

**Affiliations:** ^1^Institute of Edible Fungi, Shanghai Academy of Agricultural Sciences, Shanghai, China; ^2^Institute of Facility Agriculture and Equip, Gansu Academy of Agricultural Engineering and Technology, Wuwei, China; ^3^Institute of Technical Biology and Agriculture Engineering, Hefei Institutes of Physical Science, Chinese Academy of Sciences, Hefei, China

**Keywords:** *V. volvacea*, metabonomics, low-temperature stress, LC-MS, differential metabolites

## Abstract

Low temperature can lead to the autolysis of *Volvariella volvacea* (*V. volvacea*), hindering its growth and preservation and severely reducing its yield and quality. This autolysis of *V. volvacea* at low temperature has been reported, but a metabolomics-based investigation of the underlying mechanisms of the *V. volvacea* response to low temperature has not been reported. Therefore, this study aimed to explore the changes, levels and expression patterns of *V. volvacea* metabolites at low temperature. To understand the metabolic differences within *V. volvacea*, two strains with different levels of low-temperature tolerance were treated in an ice bath at 0°C for 2, 4, 8, and 10 h, while the blank control group was treated for 0 h. Metabonomics analysis was adopted to study the changes in *V. volvacea* in response to low temperature and the differences between the two different strains. Metabolic curves were analyzed at different time points by high-performance liquid chromatography-mass spectrometry (HPLC-MS). A total of 216 differential metabolites were identified and enriched in 39 metabolic pathways, mainly involving amino acid metabolism, carbohydrate metabolism, the TCA cycle, energy metabolism, etc. In this paper, we report the metabonomic analysis of *V. volvacea* in response to low temperature and compare the differences in metabolite expression between the low-temperature-resistant strain VH3 and the low-temperature-sensitive strain V23. Finally, the putative low-temperature resistance mechanism of VH3 is revealed at the metabolic level. This study provides a theoretical basis for revealing the regulatory mechanism of low-temperature resistance in *V. volvacea* and for future molecular breeding efforts.

## Introduction

*Volvariella volvacea* (Bull. ex Fr.) Sing. is a typical tropical and subtropical edible fungus species and one of the major edible fungi produced in China. Mushrooms of this species are nutritious and delicious (Wang et al., 1992) and are also one of the most popular fresh mushrooms among consumers. *V. volvacea* is rich in protein and shows fast growth and development and a high respiratory intensity. Under certain conditions, the caps of the mushrooms can easily open after harvest, reducing their edible value (Zhu et al., 2010). At the same time, because *V. volvacea* is a kind of high-temperature edible fungus that is sensitive to temperature change, it is usually hydrated and autolyzed at 0°C∼5°C (Chen et al., 1995; Yang et al., 2002), which accelerates its decay, making it soft and liquefied and leading to low-temperature autolysis (Qiao, 2008). Therefore, the development of the *V. volvacea* industry has been greatly limited. At present, the phenomenon of low-temperature autolysis of *V. volvacea* has been reported, but its specific mechanism has not been clearly explained. Therefore, research into the mechanism underlying the *V. volvacea* response to low temperature is urgently needed.

Metabolomics and metabonomics are concepts proposed by Nicholson et al. (1999) and are emerging branches of systematic biology, similar to genomics, transcriptomics and proteomics (Kong et al., 2014). Metabolomics aims to quantitatively study the multiple dynamic responses of living organisms to external stimuli, pathophysiological changes and the metabolites produced in their bodies caused by their own gene mutations (Nicholson et al., 1999). Metabonomics can be used to detect as many metabolites as possible in the samples. Explorations at the metabolic level provide an unbiased approach to the study of biological processes. Such approaches can also be used to analyze specific quantities or types of metabolites with subjective purposes. The main analytical techniques used in metabonomics include gas chromatography (GC), liquid chromatography (LC), mass spectrometry (MS), and nuclear magnetic resonance (NMR) (Ni et al., 2014). Based on different chromatographic mobile phases, the methods can be divided into GC-MS and LC-MS, and as an analytical technique, non-targeted metabonomics is used to determine the nature and relative quantities of metabolites in biological systems as much as possible, reflecting the total metabolite information to the greatest extent. Because there are many types of small and medium-sized metabolites in biological samples, with a large polarity span and large dynamic range, GC-MS technology has become the most important tool for metabonomics research. LC-MS is a series-based analysis platform with high-performance LC (HPLC) as the separation system and high-resolution MS as the detection system. Compared with GC-MS techniques, LC-MS is more suitable for the analysis of metabolites that are difficult to volatilize or have poor thermal stability. Ultra-high-performance LC (UHPLC) via a column packed with 1.7-μm ultrafine particles has improved analysis speed over traditional HPLC along with several improvements in sensitivity and resolution (Plumb et al., 2005). At present, the combined application of UHPLC and quadrupole time-of-flight (Q-TOF) MS is widely used in metabonomics research. Hydrophilic interaction LC (HILIC) employs a kind of chromatographic column specially developed for highly polar metabolites. This method has been widely used and is valued because of its complementary selectivity with reversed-phase LC (RPLC). Studies have shown that HILIC-ESI (±)-Q-TOF MS can provide the maximum amount of information on central carbon cycle metabolism (Ivanisevic et al., 2013). The non-target metabonomics analysis process based on UHPLC-Q-TOF MS generally includes sample pretreatment, metabolite extraction, LC-MS full-scan detection, data preprocessing, statistical analysis and differential structure identification.

In this paper, HILIC UHPLC-Q-TOF MS combined with a data-dependent collection method was used to perform full-spectrum analysis (Benton et al., 2015) and explore changes in the physiological metabolism of *V. volvacea* in response to low-temperature stress for different durations, providing new information for further studies on the mechanism of *V. volvacea* autolysis at low temperature and a theoretical basis for the genetic engineering of cold resistance in *V. volvacea*.

## Materials and Methods

### Strains for Testing

The V23 strain of *V. volvacea* used in this experiment is a high-yield strain that is sensitive to low temperature. Strain VH3 was induced from V23 and has stronger resistance to low temperature than V23. Both were provided by the Strain Preservation Center of the Edible Fungi Institute of the Shanghai Academy of Agricultural Sciences.

### Treatment and Collection of *V. volvacea* Mycelia

The V23 and VH3 strains were inoculated onto a potato dextrose agar (PDA) plate (20 mL) and cultured in an environmentally controlled incubator at 32°C for 4 days, and then, 10 mL of the substance was extracted by a pipette gun to an Erlenmeyer flask containing potato dextrose broth (PDB) (100 mL) for further culture at 32°C and 150 rpm for 5 days.

After the mycelia formed, the Erlenmeyer flask was transferred into an ice bath at 0°C and treated for 2, 4, 8, and 10 h, while the control group was treated for 0 h. The mycelia were filtered by a sterile non-woven cloth on a laminar flow cabinet and washed with sterile water several times. After the water was absorbed with sterile absorbent paper, the mycelia were collected into a sterile centrifuge tube. Seven duplicate samples were taken for each treatment. Each sample weighed approximately 0.5 g. All samples were placed in 7 tubes separately (7 biological repetitions were required; subpackaging can reduce the influence of repeated freezing-thawing), frozen in liquid nitrogen quickly, and then stored in a refrigerator at −80°C for later use.

Quality control (QC) samples were prepared as follows: Samples were mixed in equal amounts. QC samples were used to determine the state of the instrument and balance the LC-MS system before sample feeding and to evaluate the stability of the system over the whole experimental process (Want et al., 2010).

### Determination of the Physiological Indexes of *V. volvacea* at Low Temperature

#### Determination of Relative Conductivity

Mycelium balls placed in an ice bath (0°C) at different time points were put into an Erlenmeyer flask with the addition of 30 mL of ultrapure water, and the Erlenmeyer flask was placed in a shaker at 150 rpm and 15°C for 24 h and then removed to measure E1. Then, the mycelia were inactivated at high temperature and placed in a shaker under the same conditions for another 24 h to measure E2. The relative conductivity E was calculated according to the formula E(%) = (E1-E0)/(E2-E0), where E0 refers to the conductivity of ultrapure water.

#### Determination of Changes in MDA Content

The malondialdehyde (MDA) content was determined by thiobarbituric acid colorimetry as described by Heath and Packer (Heath and Packer, 1968).

### LC-MS Analysis

#### Sample Pretreatment Method

Each *V. volvacea* mycelium sample was removed. Approximately 60 mg of sample from each group was weighed and homogenized with the addition of 200 μL of water. Then, 800 μL of methanol/acetonitrile (1:1, v/v) was added, and the mixture was centrifuged for 30 s. Thirty minutes of ultrasonication was applied to the sample mixtures twice at low temperature, and the protein was precipitated after incubation for 1 h at −20°C. Then, after centrifugation at 13000 rpm and 4°C for 15 min, the supernatant was removed, freeze dried, and stored at −80°C for later use.

#### Chromatographic Conditions

Samples were separated by using an Agilent 1290 Infinity LC UHPLC HILIC chromatographic column. The column temperature was 25°C, the flow velocity was 0.3 mL/min, and the sample size was 2 μL. The mobile phase compositions were as follows: A: water+25 mM ammonium acetate+25 mM ammonia water and B: acetonitrile. The gradient elution procedure was 0–1 min at 95% B. The gradient program used was as follows: 1–14 min, B changed linearly from 95% to 65%; 14–16 min, B changed linearly from 65% to 40%; 16–18 min, B remained at 40%; 18–18.1 min, B changed linearly from 40% to 95%; and 18.1–23 min, B remained at 95%. The samples were placed in the autosampler at 4°C during the entire process. To avoid the influence of the fluctuation of the detection signal from the instrument, a random sequence was adopted for continuous analysis of the samples. QC samples were inserted into the sample queue to monitor and evaluate the stability of the system and the reliability of the experimental data.

#### Q-TOF Mass Spectrum Conditions

The positive ion and negative ion modes of electrospray ionization (ESI) were used for detection in this study. After separation by UHPLC, mass spectrum analysis was conducted for samples by using a Triple TOF 5600 mass spectrometer (AB SCIEX). ESI source conditions after HILIC chromatographic separation were as follows: ion source gas 1 (Gas 1): 60, ion source gas 2 (Gas 2): 60, curtain gas (CUR): 30, source temperature: 600°C, ion spray voltage floating (ISVF): ± 5500 V (in positive and negative modes), TOF MS scan m/z range: 60–1000 Da, product ion scan m/z range: 25–1000 Da, TOF MS scan accumulation time: 0.20 s/spectrum, product ion scan accumulation time: 0.05 s/spectrum. A two-stage mass spectrum was obtained by information-dependent acquisition (IDA), and high-sensitivity mode was adopted, with a declustering potential (DP) of ± 60 V (in positive and negative modes) and collision energy of 35 ± 15 eV. The IDA settings were as follows: exclusion of isotopes within 4 Da, candidate ions to monitor per cycle: 6.

### Data Processing

The original data were converted to mzXML format by ProteoWizard. Then, peak alignment, retention time correction and peak area extraction were performed using the XCMS program. The structures of the metabolites were identified by exact mass number matching (<25 ppm) and second-level spectrum matching with an internally built database. For the data extracted by XCMS, the ion peak with total group >2/3 was deleted.

Isotopes and internal standards were removed from the data sets. All ions were then normalized to the total peak area of each sample in Excel 2007 (Microsoft, Redmond, WA, United States) to achieve the minimum relative standard deviation (RSD). Metabolite ions with an RSD% less than 30% were used for further data processing. The positive data were combined with the negative data to form a combined data set, which was imported into SIMCA-P 14.1 (Umetrics, Umea, Sweden) for mode identification. After being preprocessed by Pareto-scaling, multidimensional statistical analysis was performed for the data, including unsupervised principal component analysis (PCA) and supervised (orthogonal) partial least square discriminant analysis (O)PLS-DA, to visualize the metabolic alterations among experimental groups after mean centering and unit variance scaling.

According to the Variable Importance for the Projection (VIP) values from the OPLS-DA model, we measured the influence intensity and explanatory ability of the expression patterns of each metabolite for the classification and discrimination of each group of samples and further examined the differential metabolites with biological significance.

If the VIP value of a metabolite was more than 1, the metabolite had a major contribution to the separation of sample groups in the OPLS-DA model. Therefore, we assumed that the variables that had a significant contribution to between-group sample separation through selection based on VIP values > 1 were appropriate for use in between-group discriminations. Based on the displacement test, an overfitting test was conducted for the OPLS-DA model. Therefore, the default 7-round cross-verification method was adopted. A total of 1/7 of the samples in each round were excluded from the mathematical model to prevent overfitting as described by Commisso et al. (2016).

### Screening of Significantly Differential Metabolites

The differential metabolites were selected based on their VIP values from the OPLS-DA model, and the combination of *P* values was obtained from the two-tailed *t*-test of the statistical variable influence threshold and standardized peak area (Fragallah et al., 2018). VIP values based on the OPLS-DA model were used to measure the influence intensity and explanatory ability of the expression pattern of each metabolite on the classification and discrimination of each group of samples and to explore the differential metabolites with biological significance. In this experiment, taking VIP > 1 as the screening criterion, the differences among groups were preliminarily screened out. Furthermore, univariate statistical analysis was used to verify the significance of differential metabolites. Metabolite VIP values > 1 from multidimensional statistical analysis and *P* values < 0.05 from univariate statistical analysis were used to identify the metabolites with significant differences, and the metabolites with VIP > 1 and 0.05 < *P* value < 0.1 were considered differential metabolites. The differential metabolites were identified and classified by using the database established by Applied Protein Technology.

### Bioinformatics Analysis of Differential Metabolites

#### Cluster Analysis of Differential Metabolites

To evaluate the appropriateness of candidate metabolites and show the relationship among samples and differences in the expression patterns of metabolites in different samples more comprehensively and intuitively, we performed hierarchical clustering for each group of samples by using R software based on the qualitative differential expression of metabolites. This helped us accurately screen marker metabolites and study the changes in related metabolic processes.

#### KEGG Pathway Enrichment Analysis of Differential Metabolites

Pathway enrichment analysis of differential metabolites is helpful for understanding the mechanisms underlying changes in metabolic pathways among different samples. KEGG^1^ pathway analysis is commonly used. By mapping differential metabolites to the KEGG database, a metabolic pathway map containing two or more differential metabolites can be obtained. Based on the pathway analysis function of the Metabolites Biological Role (MBRole) tool, KEGG IDs of different metabolites were mapped to the KEGG database and compared with the metabolic pathway enrichment results previously described by Li et al. (2015). The parameters considered in the enrichment analysis included the ID number of the metabolic pathway, the name of the metabolic pathway, the number of metabolites involved in the metabolic pathway, the *P* value of the metabolic pathway, the false discovery rate (FDR)-corrected *P* value, and the log *P* value.

#### Cluster Analysis of the Expression Trends of Differential Metabolites

The time series analysis software Short Time-series Expression Miner (STEM) was used to cluster the metabolites with similar expression spectra according to the variation in the same strain over time (i.e., intragroup change) to infer the functions of other metabolites in the same class from those of the known metabolites in the cluster.

## Results

### Determination of Physiological Indexes

#### Change in the Relative Conductivity of *V. volvacea* at Low Temperature

**FIGURE 1:**
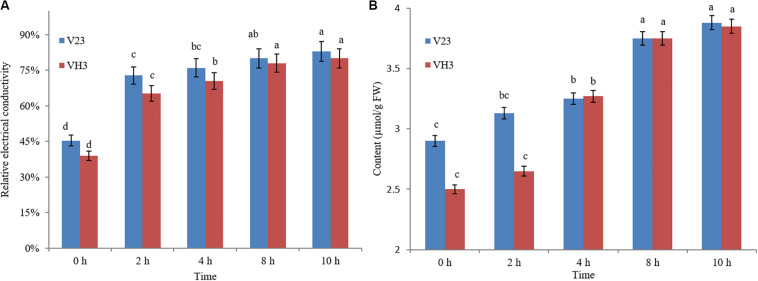
Effect of low-temperature stress on the relative conductivity **(A)** and MDA content **(B)** of *V. volvacea.* Comparison was conducted among different low-temperature treatment time points in the same strain (in V23 or VH3). The lowercase letters on bars represent the levels of significance. Means with the same lowercase letters at the top of the bar do not differ significantly (*p* < 0.05).

The changes in the relative conductivity of *V. volvacea* mycelia following treatment in an ice bath for different durations are shown in Figure 1A. With prolonged ice bath treatment time, the relative conductivity of the V23 and VH3 strains increased gradually. When treated in an ice bath for 0–2 h, the relative conductivity of the two strains increased the most, reaching 72.80% and 65.13%, respectively. This indicated that the permeability of the *V. volvacea* mycelia changed dramatically during the 2 h following the start of the ice bath treatment, which may be key information for gene-level research. At 2–10 h of ice bath treatment, the change in relative conductivity remained basically stable. After 10 h of ice bath treatment, the relative conductivity of the two strains exceeded 80%. This indicated that the selective permeability of the cell membrane of the two strains was basically lost after 10 h of ice bath treatment, which may be key information for metabolic studies. Moreover, during the ice bath treatment, the relative conductivity of the VH3 strain, which is resistant to low temperature, was lower than that of the V23 strain, which is sensitive to low temperature, which indicates that the cell membrane integrity of the VH3 strain was relatively high, possibly contributing to its low-temperature resistance.

#### Change in the MDA Content of *V. volvacea* at Low Temperature

Figure 1B shows the changes in the MDA content with time inside cells when the mycelia of *V. volvacea* strains V23 and VH3 were subjected to low-temperature stress. When strain V23 was under the low-temperature treatment, its MDA content increased gradually with prolongation of treatment, peaking at 10 h of treatment. In the first 2 h of low-temperature treatment, the MDA content was almost unchanged in VH3, but at 2–4 h of low-temperature stress, the MDA content sharply increased to 3.27 μmol/g fresh weight. From 4–10 h, the MDA content gradually increased to its highest level, which was observed at 10 h. Under 0 h of low-temperature stress, the MDA content in strain VH3 was lower than that in strain V23. At 2 h of low-temperature treatment, the MDA content in the mycelia of *V. volvacea* strain VH3 was lower than that in mycelia of strain V23; from 4–10 h, the MDA content in both strains was almost the same.

### Untargeted Metabonomics Confirmation

Untargeted metabonomics analysis can be applied to thousands of unknown metabolites. The confirmation of the method is different from the confirmation of the target object analysis method, which is more challenging. To ensure that the differences found in this study are biological differences rather than technical differences caused by measurement errors, it is necessary to perform effective QC. At present, one commonly used method worldwide is the use of mixed QC samples, where a small number of samples are reserved and then inserted evenly into the operational sequence for monitoring the stability and repeatability of the instruments and for data filtering. In this study, 2-chlorophenylalanine, an internal standard, was combined with mixed QC samples to monitor the data quality and confirm the effectiveness of the study methods. After completion of the analysis, QC sample data were used to systematically evaluate the performance of the analysis, judge the necessity for further data analysis, select metabolic characteristics, and filter out the metabolic characteristics with large variations in the QC samples, and the threshold RSD was generally 20% in the LC-MS analysis.

**FIGURE 2:**
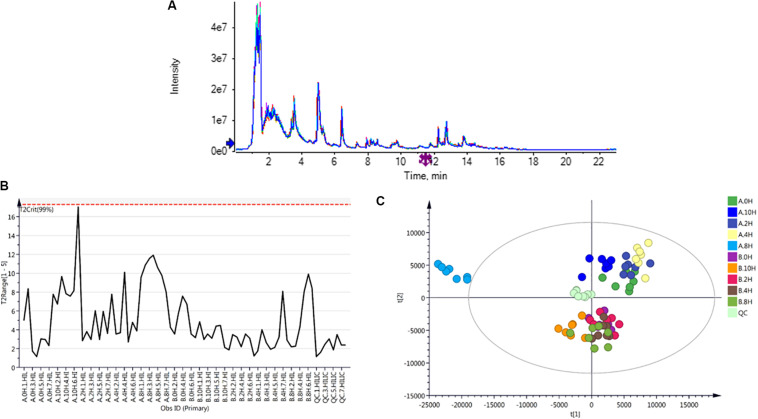
**(A)** Total ion chromatogram (TIC) of QC samples; **(B)** Hotelling’s T2 chart of samples; **(C)** principal component analysis (PCA). A represents strain VH3, and B represents strain V23. Seven biological replicates were used for each treated sample. The QC sample, in which the same amount of each sample was used for mixing, was used to test the accuracy of the experiment and to judge the reliability of the sample grouping information in unsupervised component analysis.

A comparison of overlapping spectra was conducted for the UHPLC-Q-TOF MS total ion chromatograms (TICs) of QC samples, as shown in Figure 2A. The results showed that the response intensity and retention time of chromatographic peaks basically overlapped, indicating that the variation caused by instrumental error was small throughout the experimental process. Hotelling’s T2 analysis can be used to determine whether an outlier sample exists. As shown in Figure 2B, all samples were within the 99% confidence interval. XCMS software was used to extract the ion peaks of the metabolites. A total of 6734 positive ion peaks and 5777 negative ion peaks were obtained. PCA was performed for all peaks extracted from all test samples and QC samples after Pareto scaling. As shown in Figure 2C, QC samples clustered closely in the positive and negative ion modes, indicating that this experiment had good repeatability. Through 7-fold cross-validation (7 cycles of cyclic interactive verification), a PCA model was obtained.

In conclusion, the instrument-based analysis system used in this experiment was stable, and the test data were reliable. The differences in the metabolic spectra obtained in this experiment reflect the significant biological differences among samples. The results show that the data are reliable and can be used for subsequent biological analysis.

### Data Dimension Reduction

**FIGURE 3:**
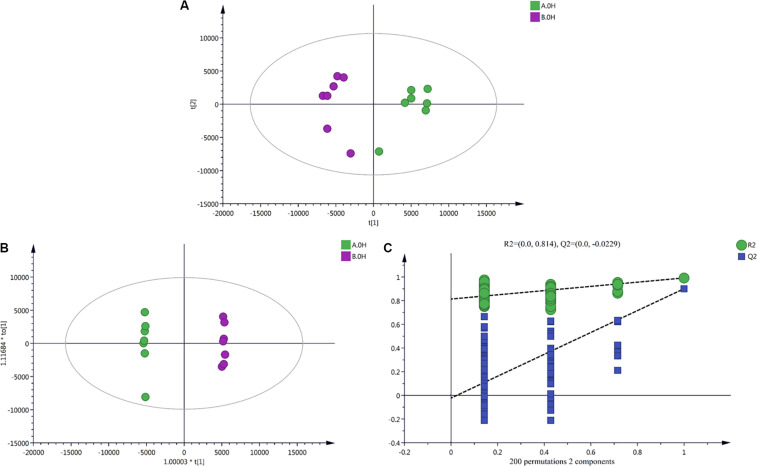
Multivariate analysis charts of *V. volvacea* samples. **(A)** PCA shot chart; **(B)** OPLS-DA analysis results; **(C)** OPLS-DA replacement inspection results. Two *V. volvacea* strains treated for 0 h were used as an example group; the results for strain V23 are shown in green, and those for strain VH3 are shown in purple. See supplementary data for the results for other treated groups at 2, 4, 8, and 10 h.

PCA results (Figure 3A) showed obvious degrees of separation among all of the mycelium samples treated in an ice bath for different durations. This reflects changes in the metabolites of the two strains (V23 and VH3) under low-temperature stress for different durations. OPLS-DA results (Figure 3B) showed that the two strains exhibited significant spectral separation at the different treatment time points. This indicated that the metabolic differences in the two strains at different treatment time points were statistically significant. A replacement inspection chart of the sample groups is shown in Figure 3C. The replacement inspection established a 200-fold OPLS-DA model by randomly changing the order of classification variable Y to obtain the R2 and Q2 values of a random model. The abscissa represents the degree of retention of replacement inspection, while the ordinate represents the value of R2 or Q2. All of these Q2 points were lower than the original Q2 points; the points are shown in blue left to right, indicating that the model was robust and reliable, without any fitting.

### Differential Metabolites in Mycelia Under Low-Temperature Stress

Among the ion peaks identified, 6734 were identified in positive ion mode, and 5777 were identified in negative ion mode. According to the metabolite peak areas, LC-MS retention times and accurate molecular weights, as well as total ion chromatography, the chemical structures of the main metabolites of *V. volvacea* mycelia were determined. A total of 547 metabolites were obtained in positive ion mode, while 446 metabolites were obtained in negative ion mode. The RSD values in positive ion mode (82.71%) and negative ion mode (80.09%) were larger than 20%, indicating good reproducibility of the metabonomics approach.

**FIGURE 4:**
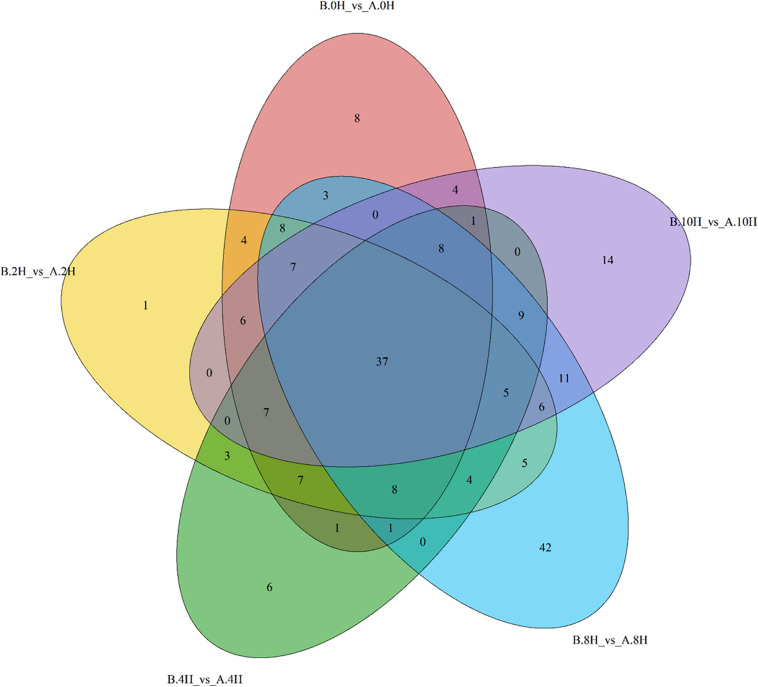
Differential metabolites of *V. volvacea* in response to low-temperature stress. Venn diagram showing the overlapping (0, 2, 4, 8, and 10 h) and stage-specific differential metabolites. A represents strain VH3, and B represents strain V23. Red: 0 h; Yellow: 2 h; Green: 4 h; Blue: 8 h; Purple: 10 h.

In this paper, with the screening conditions, namely, VIP > 1 and *P*-value < 0.05, 496 differential metabolites were preliminarily screened. Furthermore, 216 significantly differentially expressed metabolites were retained after removing redundancy and repetition; these metabolites were mainly classified as 13 different substances, such as organic acids, fatty acids, amino acids, carbohydrate metabolites, nucleotides, vitamins, alkaloids, lipids, amines, phenols, flavors, hormones, pigments and other substances. Figure 4 shows the numbers of differential metabolites of *V. volvacea* screened under 0, 2, 4, 8, and 10 h of low-temperature stress at 0°C. Among the identified metabolites, 37 metabolites were found continuously throughout the low-temperature treatment. At 0 and 2 h, there were 4 common metabolites; at 0 and 4 h, there was 1 common metabolite; at 0 and 8 h, there were 3 common metabolites; and at 0 and 10 h, there were 4 common metabolites. Moreover, 8, 1, 6, 42, and 14 metabolites were unique to the 0, 2, 4, 8, and 10 h treatment time points.

### Expression of and Change in Metabolites in Mycelia Under Low-Temperature Stress

**FIGURE 5:**
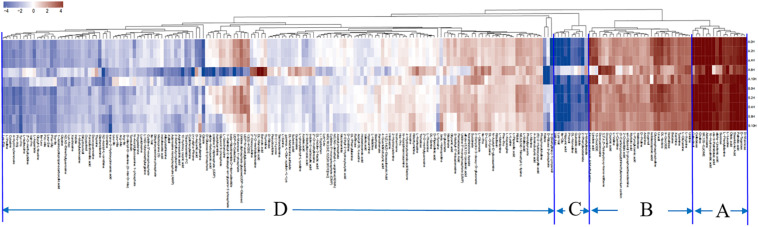
Thermogram of differential metabolite expression in two *V. volvacea* strains under different durations of low-temperature stress. According to the expression patterns and similarities in the changes of metabolites, they can be clustered into four categories. Red represents upregulation, and blue represents downregulation.

The hierarchical clustering results for significantly differential metabolites are shown in the heatmap (Figure 5). According to the expression of the metabolites in the two *V. volvacea* strains under different durations of low-temperature stress, the metabolites were divided into four categories: A–D. These four categories of metabolites can be further divided into 13 types (Table 1).

**TABLE 1:** Categories of differential metabolites of two *V. volvacea* strains under low-temperature stress for different durations.

**Category**	**Organic acids**	**Fatty acids**	**Amino acids**	**Glycometabolism**	**Nucleotides**	**Vitamins**	**Alkaloids**	**Lipids**	**Amines**	**Phenols**	**Flavors**	**Hormones**	**Pigments**	**Others**
A	0	6	2	5	1	0	1	0	0	0	0	0	0	1
B	5	1	6	2	5	0	5	1	1	0	1	0	0	3
C	2	0	6	0	0	0	0	0	1	1	0	0	0	0
D	35	3	56	16	29	3	7	0	1	0	4	1	1	4

Category (A) includes 16 compounds. These metabolites are mainly fatty acids and carbohydrate metabolites (Table 1), which were most highly expressed during the entire low-temperature treatment among the two *V. volvacea* strains. There was no significant difference in the expression levels of these metabolites between VH3 and V23.

Category (B) includes 30 compounds. These metabolites are mainly amino acids, organic acids, nucleotides and alkaloids (Table 1), which were highly expressed during the entire low-temperature treatment.

Category (C) includes 10 compounds, which are mainly amino acids and organic acids (Table 1). The expression of these metabolites was the lowest under the low-temperature treatment.

Category (D) includes 160 compounds, which are mainly amino acids, organic acids, nucleotides and carbohydrate metabolites (Table 1). The metabolites in this group were the most abundant, but their expression levels were between those of the other three categories during the low-temperature treatment. The expression levels of category (D) metabolites were lower than those of category (A) and (B) metabolites but higher than those of category (C). In category (D), some metabolites were upregulated, and some were downregulated. The metabolites in this group changed greatly during the low-temperature treatment.

In addition, it was determined from the above four groups of metabolites that there was a large difference in the changes in strain VH3 at 8 h, which was a key time point. This was also consistent with the PCA results.

### Pathway Enrichment Analysis of Differential Metabolites

**FIGURE 6:**
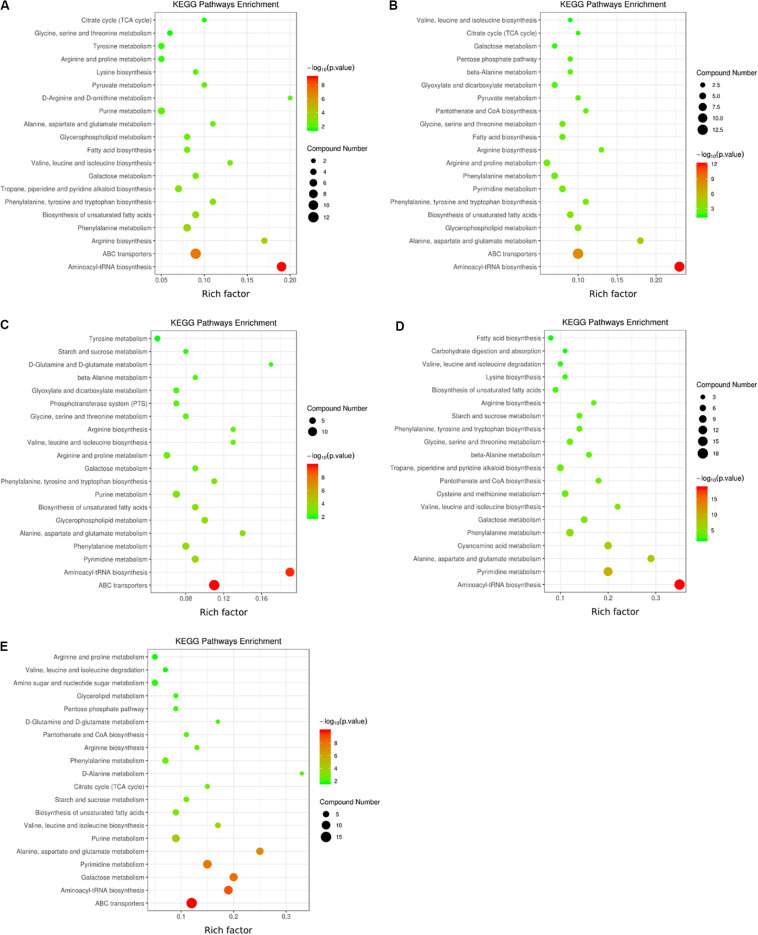
KEGG pathways in which the differential metabolites of the two strains were enriched at five low-temperature treatment time points **(A)** KEGG pathways in which metabolites were enriched at 0 h; **(B)** 2 h; **(C)** 4 h; **(D)** 8 h; and **(E)** 10 h. The color legend on the right shows the significance levels represented. Red represents an extremely significant level (*P* < 0.01), while green represents a significant level (*P* < 0.05). The circles represent the number of metabolites involved or enriched in the pathways. The enrichment factor refers to the ratio of the number of differential metabolites expressed in the corresponding pathway to the total amount of metabolites annotated in the pathway. The greater the value is, the larger the degree of concentration.

KEGG pathway enrichment analysis of differential metabolites is helpful to understand the mechanism of metabolic pathway changes in different samples. Figure 6 shows that the differential metabolites of the two *V. volvacea* strains were mainly enriched in 39 metabolic pathways during the low-temperature treatment; these pathways were mainly amino acid metabolism, carbohydrate metabolism, the TCA cycle, energy metabolism, etc. At the different intervals of low-temperature stress, the differential metabolite enrichment in the two *V. volvacea* strains was different.

The aminoacyl-tRNA biosynthesis pathway was the most significant in the initial stage (0–2 h) of low-temperature stress, followed by the ABC transporter pathway. At 4 h, the aminoacyl-tRNA biosynthesis pathway was as significant as the ABC transporter pathway. At 8 h, the aminoacyl-tRNA biosynthesis pathway was the most significant, followed by the pyrimidine metabolism pathway, while the ABC transporter pathway was not significant. At 10 h, the ABC transporter pathway was the most significant, and the other significant pathways were aminoacyl-tRNA biosynthesis, pyrimidine metabolism, galactose metabolism, and alanine metabolism. The metabolic pathways changed significantly with the duration of low-temperature stress, and the amounts of different metabolites enriched in the same metabolic pathway also changed, especially from 8 h onward, which was an inflection point for treatment time.

### Cluster Analysis of the Expression Trends of Differential Metabolites

**FIGURE 7:**
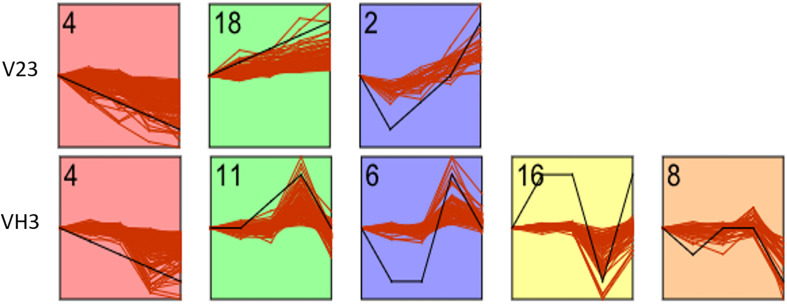
STEM analysis of differential metabolites of two *V. volvacea* strains under low-temperature stress for different durations. The Y axis represents the relative content of metabolites, and the X axis represents the duration of low-temperature stress. The inflection point in the figure is the treatment time point (0, 2, 4, 8, and 10 h). The black line represents the trend pattern. Other red lines similar to the trend of this black line would be grouped into a class. Each red line represents a metabolite. The number in each figure is the number of profiles. There are 21 profiles in total. The figure shows the selected parts of profiles with research significance.

To further analyze the dynamic trends of the metabolites of the two strains at different low-temperature treatment time points, time series analysis of the differential metabolites screened in the previous step was carried out by using STEM software. According to the expression trends of metabolites at different time points, all differential metabolites were divided into 21 profiles. As shown in Figures 3, 7 profiles were significantly enriched in strain V23 (*P*-value < 0.05), namely, No. 4, No. 18, and No. 2. Profile expression trends included 148, 70, and 26 differential metabolites; there were 5 profiles enriched significantly in strain VH3 (*P*-value < 0.05), namely, No. 4, No. 11, No. 6, No. 16, and No. 8, which included 114, 93, 68, 59, and 36 differential metabolites, respectively. See supplementary data for specific metabolites contained in each profile.

## Discussion

Temperature plays a decisive role in the growth, storage and quality of *V. volvacea* (Zhao et al., 2018, 2019a). *V. volvacea* is not resistant to low temperature, and its mycelia easily undergo autolysis, causing the fruiting bodies to wilt, become liquefied, turn brown, and rot. Low temperature directly damages the cell membranes of *V. volvacea* mycelia, resulting in changes in cell permeability and electrolyte leakage, that is, an increase in relative conductivity. Moreover, free radicals are metabolized and produced in the process of aging and injury, but their scavenging abilities are reduced, leading to an imbalance in active oxygen and membrane lipid peroxidation and finally MDA production. The accumulation of MDA is an important indicator of cell membrane system aging and damage (Lu et al., 2013; Wu et al., 2014). From these phenotypic characteristics and physiological indicators, it can be seen that the mutant strain VH3 has a stronger ability to resist low temperature stress than the original strain V23, but in general, low temperature had a considerable impact on *V. volvacea*. A continuous low-temperature environment changes the accumulation of *V. volvacea* metabolites and hinders the growth of this species.

Metabolism can reflect different states of living organisms. With the continuous improvement of metabolite detection and identification technologies, metabolic regulation in response to abiotic stress has been widely studied (Guy et al., 2008; Shulaev et al., 2008). Based on LC-MS technology, the differences in metabolite accumulation in response to low-temperature stress between two *V. volvacea* strains with different levels of cold resistance were compared. The results showed that there were significant differences in the expression patterns of metabolites between the two *V. volvacea* strains under low-temperature stress. In addition, the identified metabolites changed over the whole low-temperature treatment process. The main reason for these changes may have been the genetic background and the specificity of the two strains. The changes in metabolites showed that amino acids, organic acids, nucleotides, sugars, fatty acids, alkaloids, flavors and other substances affect the growth and preservation of *V. volvacea* under continuous low-temperature stress. The changes in metabolites caused by low-temperature stress are similar to those caused by abiotic stress in other species, especially the changes in sugars, amino acids, organic acids and other substances. The results showed that temperature stress led to a large change in sugar alcohol substances among the metabolites of *Lentinula edodes*, such as trehalose, maltose, fructose, galactose, mannitol, and amino acids, such as glycine, serine, lysine, and alanine. Under temperature stress, these metabolites were upregulated, and the stress response mechanism was initiated, which mainly involved glycolysis, the TCA cycle, energy metabolism, carbon metabolism and other pathways (Zhao et al., 2019b). Bao Yuzhuo et al. compared and analyzed the metabolites of Dongnongdongmai 1 under low temperature. The results showed that the main metabolites were sugars, amino acids, nitrogen, organic acids and nucleic acids. The levels of amino acids, such as Pro and Val, and most carbohydrates increased, which enhanced the osmotic regulation of winter wheat at low temperatures; however, a few metabolites, such as ASP, sorbitol, honey disaccharide and threitol, decreased and were involved in metabolic adaptation to low temperatures (Bao et al., 2017). In Li et al.’s study, the unsaturation of fatty acids in *V. volvacea* at 0°C was significantly reduced, especially that of phospholipids, which are important components of cell membrane structure. This indicates that *V. volvacea* may not be able to adjust its fatty acid composition to cope with low-temperature stress, eventually leading to autolytic death (Li et al., 1992). Zhao Yunfeng studied the role of cell wall metabolism in the autolysis of longan pulp after harvest. The results showed that the main metabolites involved in autolysis were protopectin, cellulose and hemicellulose (Zhao, 2005). Wu Zhiliang et al. used proteomics to study the low-temperature autolysis of the *V. volvacea* fruiting body. They believed that the mechanism of the *V. volvacea* response to cold stress was very complicated and was associated with energy metabolism, hydrolase activity, trehalose biosynthesis, amino acid metabolism, aminopeptidase activity, the MAPK signaling pathway, calcium signal transduction, reactive oxygen species metabolism, fatty acid synthesis and so on (Wu et al., 2019). This is consistent with the results of metabonomics in *V. volvacea* mycelia. This finding shows that the changes in metabolites in different species under low-temperature stress are different, which is related to the biological characteristics of the species itself; however, different species also have metabolites that jointly regulate temperature stress, mainly sugars, alcohols, amino acids, etc.

With the progression of low-temperature stress, the changes in the metabolites of *V. volvacea* were significantly different. Amino acid metabolism under low temperature manifests as changes in the relative levels of some metabolites, such as Pro and Val, which increase gradually, enhancing osmotic regulation in *V. volvacea* under low temperature; a few amino acids, such as Asp, decrease in relative abundance with prolongation of low-temperature treatment and participate in the metabolic cycle involved in low-temperature adaptation. The metabolism of carbohydrates is increased during the process of low-temperature adaptation, and the levels of most carbohydrates increase with prolonged time of low-temperature stress. Among them, sorbitol, honey disaccharide, sucralite and other substances are induced by low temperature, and due to the appearance of these soluble sugars, the osmotic adjustment ability of *V. volvacea* is improved. Nitrogen metabolism also changes significantly with increased time of low-temperature treatment. For example, the relative content of spermidine decreases first and then increases slightly under different durations of low-temperature stress; the relative levels of urea and hexanediamide increase significantly after being induced by low temperature. These changes in nitrogenous compounds aid the growth of *V. volvacea* under low temperature. Organic acids also change greatly in the process of temperature adaptation, but most of these compounds are intermediate metabolites, so it is difficult to determine their specific roles in the cold resistance process.

Comparison of the mutant strain VH3 with the original strain V23 showed that the low-temperature-resistant strain VH3, which was developed via mutagenesis, had more regulatory metabolites, was involved in more complex biological processes and had greater low-temperature response activity. Five significant time series expression trends were observed for VH3. However, V23 had only three such trends. This reflects the difference between the two strains in terms of their response to low temperature. However, there was also one group with a common expression trend between both strains. The general expression trends of metabolites, including ribose alcohol, phosphorylcholine, AMP, ornithine, glutathione, etc., decreased.

## Conclusion

Along with the phenotypic changes of *V. volvacea* under low-temperature stress, the expression and changes in metabolites during the whole treatment process can be explored by using metabonomics analysis. There were significant differences in the expression patterns of metabolites between the two *V. volvacea* strains under low-temperature stress, which preliminarily revealed the mechanism of low-temperature resistance in strain VH3. In this study, on the one hand, the dynamic changes in *V. volvacea* were studied at different time points of low-temperature stress; on the other hand, a parallel comparison between the low-temperature-resistant strain VH3 and the low-temperature-sensitive strain V23 was conducted to determine the possible mechanisms underlying the resistance to low temperature in this species. An initial metabonomic analysis of *V. volvacea* under low-temperature stress was performed, and many metabolites were identified and found to be enriched in various pathways. It is necessary to study the specific functions and regulatory pathways of key significantly differential metabolites in detail to further understand the low-temperature resistance mechanism of *V. volvacea* strain VH3.

## Data Availability Statement

The datasets generated for this study are available on request to the corresponding author.

## Author Contributions

XZ, YZ, and XS conceptualized the data. XZ performed the data curation. MC, ZL, HY, LZ, CY, and YW performed the formal analysis. YZ and XS acquired the funding and supervised the data. XZ and MC performed the investigation. XZ and ZL performed the methodology. XZ performed the project administration and wrote the original draft of the manuscript. HY, CY, and LZ performed the resources. XZ and YW performed the validation. YZ and YW wrote, reviewed, and edited the manuscript. All authors contributed to the article and approved the submitted version.

## Conflict of Interest

The authors declare that the research was conducted in the absence of any commercial or financial relationships that could be construed as a potential conflict of interest.

## Footnotes

1 https://www.genome.jp/kegg/
